# Policymaker perspectives on self-management of disease and disabilities using information and communication technologies

**DOI:** 10.1186/s12961-023-01004-7

**Published:** 2023-06-14

**Authors:** Amélie Gauthier-Beaupré, Bruno J. Battistini, Craig Kuziemsky, Jeffrey W. Jutai

**Affiliations:** 1 Interdisciplinary School of Health Sciences, University of Ottawa, Ottawa, ON Canada; 2 School of Business, MacEwan University, Edmonton, AB Canada

**Keywords:** Public Health Policy, Self-management, Aging, Health care, Qualitative research

## Abstract

**Background:**

Policies that support health self-management are malleable and highly dependent on various factors that influence governments. Within a world that is shifting toward digitalization due to pressures such as the COVID-19 pandemic and labor shortages, policymaking on older adults’ self-management of chronic diseases and disability using information and communication technologies (ICTs) needs to be better understood. Using the province of Ontario, in Canada, as a case study, the research question was What is the environment that policymakers must navigate through in development and implementation of policies related to older adults’ self-management of disease and disability using information and communication technologies (ICTs)?

**Methods:**

This study used a qualitative approach where public servants from 4 ministries within the government of Ontario were invited to participate in a 1-h, one-on-one, semi-structured interview. The audio-recorded interviews were based on an adapted model of the policy triangle, where the researcher asked questions about the influences from the different sources identified in the model. The interviews were later transcribed and analyzed using a deductive-inductive coding approach.

**Results:**

Ten participants across 4 different Ministries participated in the interviews. Participants shared insights on various aspects of context, process and actors that help shape the current content of policies. The analysis revealed that policies, in the form of programs, services, legislation and regulations, are the result of collaborations and dialogue between different actors and get developed and implemented via a set of complex government processes. In addition, policy actions come from a plethora of sectors which all get influenced by several predictable and unpredictable external pressures.

**Conclusions:**

The environment for policymaking in the government of Ontario regarding older adults’ self-management of disease and disability using ICTs is one that is mostly reactive to external pressures, while organized within a set of complex processes and multi-sectoral collaborations. The present research helped us to understand the complexity of policymaking on the topic and highlights the need for increased foresight and proactive policymaking, regardless of which governments are in-place.

**Supplementary Information:**

The online version contains supplementary material available at 10.1186/s12961-023-01004-7.

## Background

Since the start of the COVID-19 pandemic, policymaking environments around the world have been challenged and had to adapt to keep populations safe from infectious threats [1]. In their rapid responses, governments made several critical decisions on ways to deliver programs and services to the population. In some countries, such as Belgium, France and Canada, governments digitalized processes and increased the use of technological tools for health care delivery to ensure the safety of populations [2]. For Canada, this included using digital solutions for contact tracing [2]. At a provincial level, the government of the province of Ontario in Canada recognized the benefits of digital technology, such as information and communication technologies (ICTs), in meeting the needs of Ontarians on a variety of levels including healthcare [3]. The digitalization shift during the COVID-19 pandemic led to major changes in programs and services impacting several segments of the population, such as those living with chronic health conditions and disabilities [4]. While these shifts had some positive impacts, they also disrupted several aspects of care management [5]. For instance, COVID-19-related public health restrictions had detrimental effects on health conditions and mental health of individuals [6, 7] and also caused disruptions in healthcare delivery in health systems globally [8]. For this reason, there is a need to ensure that policymakers are well equipped to adapt and respond to emergencies in a way that considers the needs of people living with chronic diseases and disabilities. While COVID-19 remains a major influencing factor in policy decisions, other contextual factors such as political regimes, the number and type of actors involved, and policy development and implementation processes all play a role in the formulation of policies [9].

In this study, we conducted a comprehensive analysis of current policymaking related to older adults’ self-management of disease and disability using ICTs by identifying factors that shape policy development and implementation. While COVID-19 increased pressure on governments to consider digital supports in the delivery of programs and services [3], ICTs were already being used for—and by—older adults to support self-management [10]. In fact, the role of ICTs in supporting self-management by improving health decision-making and behaviours is now well known across the world [11]. For older adults specifically, self-management using ICTs presents opportunities for increasing physical and social well-being, improving health outcomes [12–14], and reducing growing demands on health services [15]. However, implementation of such technologies has been slow and inconsistent in many countries.

For the Canadian context specifically, there is limited knowledge on policymaking related to older adults’ self-management of disease and disability using ICTs, and more specifically, which factors shape policy decisions on this issue. Prior research identified that chronic disease self-management, as a concept, is included in most Canadian provincial policies (editor’s note: healthcare is of provincial jurisdiction in Canada) [16], but when coupled with technology, the number of policies are scarce [17]. Using the province of Ontario, Canada as a case study, this work aimed to get a better understanding of policymaking and the influences that shape such policies. The study is based on an adapted model of policy analysis by Walt and Gilson [9] where we critically analyzed factors of the content, actors, process and context to better understand the government of Ontario’s policymaking on older adults self-management of disease and disability using ICTs.

### Aim of the study

The aim of this study is to describe the environment for policymaking in Ontario related to older adults’ self-management of disease and disability using ICTs. This includes the analysis of factors that influence policy development and implementation within a federated system. Perspectives and experiences of policymakers were gathered on various aspects of policymaking (context, actors, and process) to identify influences on policy decisions.

More specifically, the research question for this study was: *What is the environment (including context, actors and process) that policymakers must navigate through in the development and implementation of policies related to older adults’ self-management of disease and disability using information and communication technologies (ICTs)?*

Research sub-questions include:

1. What are current policies and policy actions for older adults’ self-management of disease and disability using ICTs?
2. Who is involved in the creation, implementation and evaluation of these policies?
3. How are these policies developed, implemented and evaluated?
4. What contextual factors impact these policies?
5. How is the current policy environment responding to innovation and advancements ICTs that support self-management?

## Methods

### Study design

**Fig. 1:**
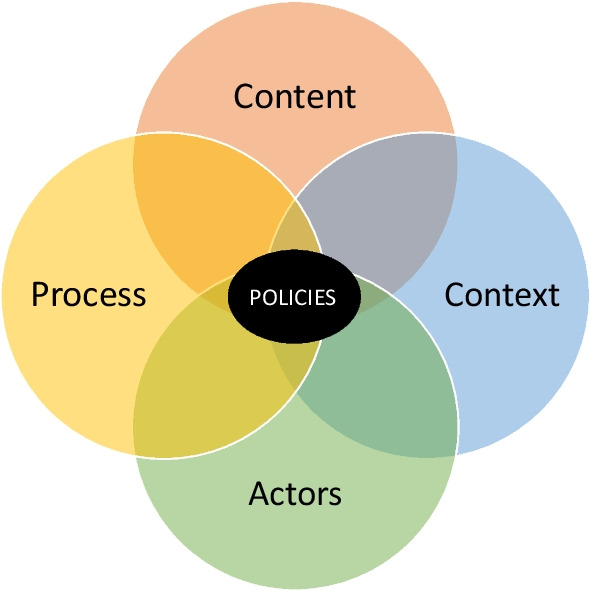
An adapted model for health policy analysis. Adapted from [9: p. 354]. Copyright 1994 by Oxford University Press and from [17: p. 5]. Copyright © 2023, A. Gauthier-Beaupré et al. Creative Commons Attribution 4.0 International License: http://creativecommons.org/licenses/by/4.0/

This qualitative study is based on an adaptation of Walt and Gilson’s [9] policy analysis model called the policy triangle (Fig. 1). In this model, the authors emphasize the interrelationship between concepts of content, actors, process and context, and propose that they allow for a comprehensive analysis of policies [9]. Figure 1 visually represents an adaptation of this model to demonstrate how each of the concepts cannot exist without the other and must all be considered in the conduct a comprehensive analysis of policies [9]. For this study, the topic of interest was the policy actions from the government of Ontario on older adults’ self-management of disease and disability using ICTs from the perspective of public servants. This study used semi-structured interviews to gather information about each of the concepts of the policy triangle and analyzed particularities about processes, actors, and context that shape current approaches to policymaking.

### Participants

This study invited participation of public servants working in the government of Ontario, Canada. We targeted specific divisions and branches within multiple ministries of the government of Ontario if they performed work related to policies, programs or services for older adults, people with disabilities, health technologies, or collaborated with units that conduct work in these areas.

The eligibility criteria were the following:

- Employed by the government of Ontario
- Working in the (i) Ministry of Health, (ii) Ministry of Children, Community and Social Services, (iii) Ministry for Seniors and Accessibility or (iv) Ministry of Economic Development, Job Creation and Trade.
- Occupied current position for at least 1 year
- Able to understand and speak English
- Policymaking experience on files that concern older adults and technology or disability and technology

### Recruitment

Potential participants were invited to participate in the study through a phased approach. In the first phase, we invited public servants working at the highest levels such as deputy minister, assistant deputy minister, director, manager, and executive lead. At the onset, the research team read through ministry mandates to select ministries that would most likely conduct work with the target population group. As a result, we included three ministries (Ministry of Health, Ministry of Children, Community and Social Services, and Ministry for Seniors and Accessibility). To further identify participants, we retrieved contact information of potential participants via the online Government of Ontario employee and organization directory (INFO-GO). Invitation emails were then sent to all of the individuals identified in preliminary screening. In phase 2, we invited public servants working at lower levels such as senior program or policy advisors and senior program coordinators, all of which worked under the lead of previously invited individuals. In phase 3, we used snowball sampling to identify additional participants. Existing participants shared the information of the study with additional potential participants and also shared the names of other key informants with the researchers. As a result, a fourth Ministry was added in the eligibility criteria, the Ministry of Economic Development, Job Creation and Trade.

### Data collection

Data was collected during 1-h semi-structured interviews (see Additional file 1) between public servants working in the government of Ontario and the principal investigator. This was achieved through a videoconferencing platform to comply with the public health measures in place during the time of data collection.

At the beginning of the interviews, the interviewer allocated enough time to establish rapport with the interviewee and go through the consent form to remind participants of the goals of the interview. The researchers obtained verbal consent from participants and documented it using audio-recording. During the interviews, the researchers asked question about participants’ policymaking activities using questions in the four domains of the policy triangle.

Between October 1, 2021, and January 31, 2022, we conducted a total of 10 semi-structured interviews virtually, via Microsoft Teams, with policymakers from the above-cited ministries of the government of Ontario. Two participants worked for the Ministry of Health and performed tasks related to programs, five participants worked for the Ministry for Seniors and Accessibility and worked either on policy or program-type activities, one participant worked for the Ministry of Children, Community and Social Services in service delivery, and two participants worked for the Ministry of Economic Development, Job Creation and Trade in policy, program and service delivery.

### Data analysis

Prior to data analysis, the audio-recordings were transcribed verbatim and organized into the qualitative data analysis software NVivo (released in March 2020). Data were coded using a directed content analysis approach [18] which uses a hybrid deductive and inductive coding modality. As per this coding approach, we identified a preliminary coding structure to guide data analysis. The predefined coding structure was composed of the policy triangle concepts [9] to permit coding for actors, content, context and processes of policymaking components. Subsequently, codes were added to the coding structure using an inductive approach. Content analysis allowed for further interpretation and organization of codes into themes (Table 1).

**Table 1:** Selected codes, categories, and themes for data analysis

Codes (deductive)	Categories (inductive)	Themes (inductive)
Content	Policy	Diversified foci and form
	Program	
	Service delivery	
Context	Provincial political agenda	Organisation of the Canadian system
	Constitutional (Federal, Provincial and Territorial (FPT) relations)	
	Knowledge exchange activities and events	
	Emergencies (i.e., COVID-19)	Influences from unpredictable external pressures
Process	Idea generation	Hybrid and malleable information exchange via a collaborative process
	Policy development	
	Implementation	Cautious and adapted implementation
	Evaluation	Performance measurement via various tools and techniques, and quality improvement via innovation
Actors	External partners	Multi-sectoral collaboration with a set of relevant and knowledgeable actors
	Internal partners	

In this study, we used a set of techniques to ensure rigour and trustworthiness of the results. As per Korstjens and Moser, we used quality criteria to ensure credibility, transferability, dependability, confirmability and reflexivity [19]. To ensure credibility, we performed data triangulation by collecting experiences from public servants of different levels, areas, and expertise. To ensure transferability, we provided detailed descriptions about the context for the study, including the sampling strategy, the timeframe for data collection, and demographic information of participants. For dependability, we engaged with an external evaluator who reviewed and approved the proposed approach for data collection and analysis. Finally, we ensured reflexivity by supplementing interview transcripts with field notes which include reflexive accounts from the interviewer.

### Ethics

This study received ethical approval from the University of Ottawa’s Health Sciences and Sciences Research Ethics Board (Ethics File Number H-07-20-5555). All semi-structured interviews were conducted via a secure videoconferencing platform with participants who agreed to participate and received approval from senior management (as needed). The researchers provided participants with consent forms that highlighted details about the study, the risks and benefits of participating, the measures to ensure confidentiality and anonymity, the conservation of the data, and the voluntary nature of the participation. All participants provided verbal consent which was audio-recorded for documentation purposes.

## Results

In the government of Ontario, a variety of policy work relating to older adults’ self-management of disease and disability using ICTs is underway. These include (i) implementation and oversight of legislation and regulations, (ii) program development, implementation and delivery, and (iii) the provision of services to communities and businesses in Ontario.

**Fig. 2:**
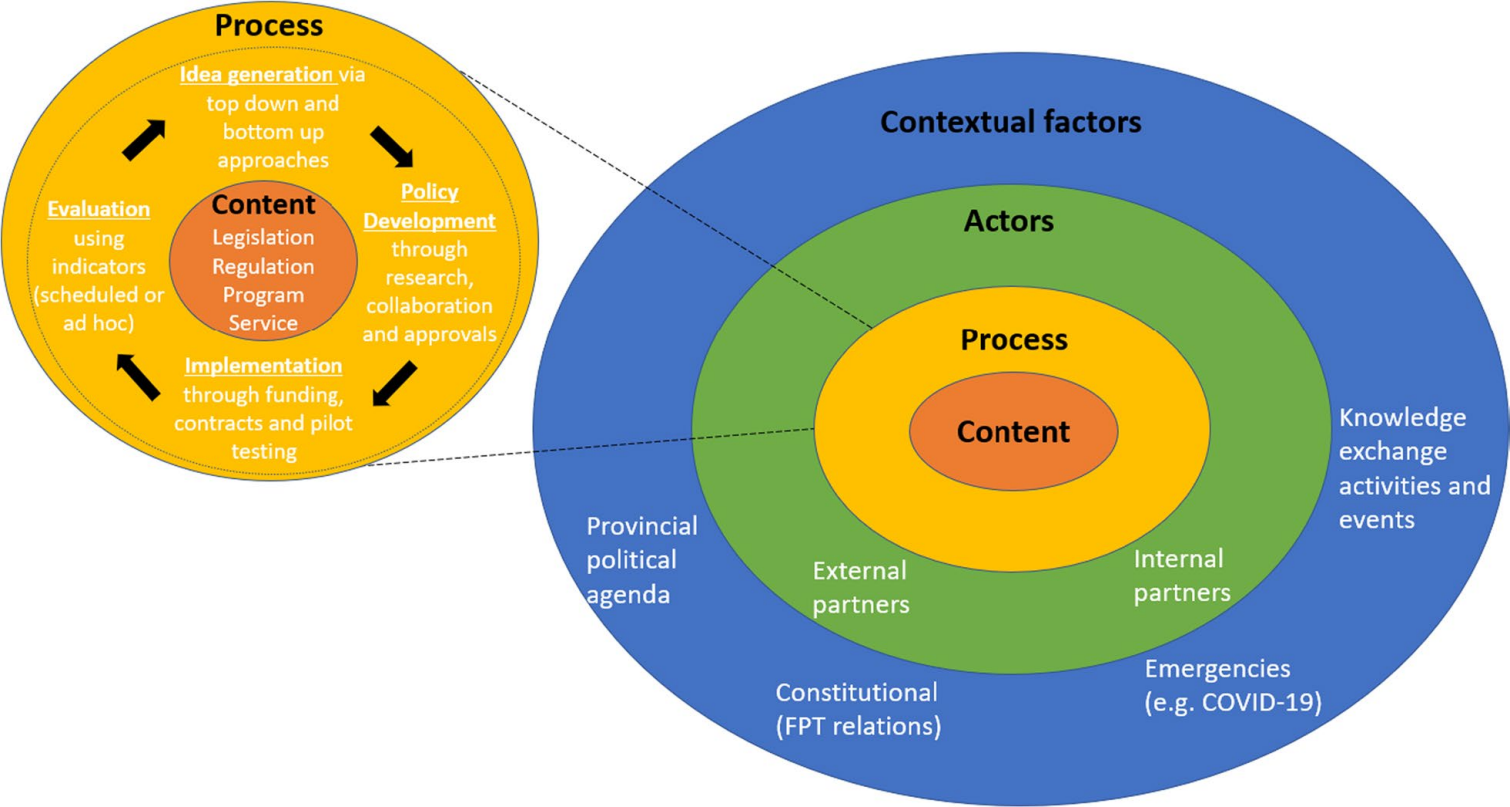
Framework for policymaking on older adults’ self-management of disease and disability using ICTs

Based on the policy triangle by Walt and Gilson [9], some key characteristics of this policy work stand out. All work from the government on self-management policy is a result of the intersection between contextual factors, actors involved in the policy, and choices in process of policymaking. As a result, we organized key components that influence policymaking in the area of older adults’ health self-management of disease and disability using ICTs in a multi-layered framework (Fig. 2).

The results of this study are presented using each of the circles of Fig. 2, and further defined using the emerging themes for each category. This approach aligns with data collection and analysis whereby the policy triangle domains guided discussions and coding strategies. While the results are presented into separate sections, the findings point to the strong intersection and interrelation between each of the components of the policy triangle, as shown in Fig. 2.

### Content

The center of Fig. 2 presents the content of policies on older adults’ self-management of disease and disability using ICTs in Ontario. The main characteristic of the policies is that they are extremely diversified in form and foci. During the interviews, participants reflected on the various ways in which they engage in policymaking which include the creation and oversight of legislation and regulations, and the delivery of programs and services (through direct funding or service to communities and businesses).

#### Diversified foci and form

In Ontario, legislation and regulations are overseen by different ministries and impact health self-management in various ways. Participants mentioned the following Acts: (1) the Retirement Homes Act (2010) for its relation to care and care management within the retirement home setting, (2) the Seniors Active Living Centres Act (2017) and its link to aging in place and caring for oneself within this environment, (3) the Accessibility for Ontarians with Disabilities Act (AODA) (2005) and the requirements it sets out to enable Ontarians with disabilities to participate in society free of barriers, and (4) the Information and Communication Standards within the AODA (2012) for the criteria it sets out for accessibility within technology, such as websites. The legislation and regulations identified by participants are operationalized through a series of programs and services that take either a disease-specific approach or support chronic disease management more generally, with a focus that has broadened over time.

One major program of the Government of Ontario is the former telehomecare program coordinated by the Ontario Telemedicine Network (OTN) [20, 21]. This program equipped individuals with advanced communications and information technology, such as tablets, enabling them to participate in care monitoring alongside health care professionals [20, 21]. The program evolved to be less restrictive, include more clinical domains and where funding is delivered through Ontario Health Teams (OHT), a group of providers and organizations that “provide a new way of organizing and delivering care that is more connected to patients in their local communities” [22] and support implementation of technologies in different clinical settings. In one of the interviews, a participant mentioned that the newer version of the program was less restrictive than the previous toward the age of participants, but remained specific to people living with a chronic disease and that have a certain degree of ability with technology. Participants also pointed to another program of interest through OHT that allows to lend technologies to patients who may not have access to it (i.e., due to prices of technologies). Additionally, participants mentioned home care services, where partner organizations deliver home and community care supports as an indirect service from the government of Ontario in support of self-management activities, which includes improving use of digital technologies with the goal of increasing access to care.

The legislations and regulations mentioned above help to profile the government of Ontario’s actions toward supporting older adults self-manage their conditions using ICTs. In addition, participants noted that their support role for other areas of government also increased their effort in that direction. They mentioned that they are sometimes asked to provide an ‘older adult lens’ in the development of policies for other sectors. This was the case in an example from the Ministry for Seniors and Accessibility in response to a request by the Ministry of Transportation:

We are in a sort of stewardship role; we work with various ministries on their own initiatives. […] The Ministry of Transportation is working right now around an action plan to prepare for connected vehicles and automated vehicles and that sort of technology, and so we're working very closely with them to understand both the impacts for seniors, for older adults, as well as people with disabilities. (P7)

Finally, certain participants mentioned the government’s work with businesses (within the private sector) on the topic of technology. They indicated that this work lies at the interface between health, life sciences and technology. As such, the government of Ontario supports businesses in ways that indirectly influence accessibility and availability of technologies that support self-management within the context of Ontario.

Overall, policies for older adults’ self-management of disease and disability using ICTs in Ontario are enacted as legislations, regulations, programs and services, and may have direct or indirect impact this segment of the population. The existence of such policies alone, however, do not suffice. As demonstrated in Fig. 2, each of the domains of the framework intersect to form the full breadth of policy actions on the topic. The following sections will point to the permeability of each domain with the others.

### Process

As identified by the participants, the process for policymaking used by the government of Ontario is divided into 4 steps: (1) idea generation, (2) policy development, (3) implementation, and (4) evaluation. Each of which can be thematically categorized. During the interviews, participants often presented steps 1 and 2 as intertwined together in the policymaking process, so we will present these 2 steps together.

#### Idea generation and policy development: hybrid and malleable information exchange via a collaborative process

Before policies are formulated and implemented, there is an idea generation phase. This phase is characterized by a series of information exchange and brainstorming mixed with some policy development components. It is hybrid because ideas come both from the bottom-up (working level) and from the top-down (management level). Participants emphasized that policy ideas were the result of lessons learned, where new and better ways to conduct work were prioritized. The interplay between bottom-up work (generated from lessons learned or pressured via advocacy from external bodies) and direction from broader government strategic priorities were the main drivers for idea generation and policy development.

In the idea generation and policy development phases, participants mentioned the creation of expert committees to explore certain issues of importance. The expert committees were said to make recommendations on policy options to support the policy development phase after a series of consultations and meetings. Participants mentioned that the exchange of ideas between different levels of policymakers or experts had significant impacts on the development of policy. Consistently, participants mentioned that the process of idea generation and policy development involved consultations with higher levels of management to obtain buy-in and ensure alignment with the broader mandate of the ministry. When asked about how innovative ideas are explored by government officials, participants shared the importance of the hierarchy. They described the process as one that requires refinement and navigation within the hierarchical layers of government until it reaches the higher levels of decision-makers.

#### Implementation: Cautious and adapted implementation

The implementation of policies varies significantly across the different sectors. For programs or services, the main implementation method is through contracts with delivery partners where external organizations or delivery partners offer services to citizens.

For legislations and regulations, implementation is in the form of consultation services or liaison from the ministry. Participants mentioned their role in supporting companies or businesses in interpreting legislation and regulations or giving advice to support company development and growth. For example, the private business sector was described as an important ‘solution-provider’, while the government entity is an enabler to achieving the desired outcomes and solutions. In addition, participants mentioned their role as navigators to support companies and businesses access appropriate programs to support their growth.

Another emerging characteristic of policy implementation is pilot testing. Many participants discussed the use of pilot projects to test the feasibility and assess the benefits of new programs or services before scaling up to larger programming. When asked about innovating in policies, participants mainly described the need to pilot test and prototype the new ideas. This would allow for better risk management in the case of programs that fail to provide the expected outcomes.

#### Evaluation: Performance measurement via various tools and techniques, and quality improvement via innovation

Finally, programs or services of the government of Ontario have an evaluation component in the form of performance measurement. These are conducted using a series of tools and techniques which lead to quality improvement and innovation. They are usually built into the programs and services and serve as an accountability mechanism with external delivery partners. In other instances, participants talked about using indicators and metrics to measure the success of programs. This data was said to further support funding requests made to Treasury Board and provided clear demonstration of the impacts of the programs in reaching its planned outcomes and objectives.

As for the timing of evaluation activities, there seem to be some variation across ministries. Participants mentioned that older programs had not been evaluated often, but recent efforts increased the number of assessments, especially for newer programs. For some programs, the collection of indicators was done as often as on a monthly basis. Besides this recent effort, participants shared that it was not uncommon for programs to only get evaluated every several years due to the high number of programs that need to be assessed.

Finally, several mechanisms support the conduct the evaluations. Participants mentioned that, previously, the norm was to contract external consultants to perform the evaluations, but that there was a recent shift toward conducting internal examinations and evaluations. The rationale explaining this shift is that work from external consulting companies were found to be of low quality which increased workload for public servants, often requiring them to rewrite the reports. It remained common, however, to see evaluations getting conducted externally by the organizations or partners implementing the programs, but still getting reviewed by government of Ontario employees. Ultimately, the goal with the internal reviews were to ensure that the delivery of programs followed a predetermined direction.

As mentioned previously, evaluations are often used to support requests for increased funding and provide a mechanism to improve and innovate. This was discussed significantly by P4 who talked about monitoring and the need to improve services by innovating:

Our days are spent, sort of, problem solving. Doing a lot of what we call service design, so analyzing existing services that are in place and looking for opportunities for improvement. […] So basically, just now that we're sort of “Post COVID”, how do we recover from that period of time? And how do we look at what's next for us, how do we continue? You know, COVID wasn't all bad, right? It increased our digital uptake at lots of things. So how do we now continue on this trend of moving towards digital services, but also making sure that we're supporting? (P4)

In this previous example, the participants also point to the role of the external pressures, such as the COVID-19 pandemic and their impact on policymaking processes. This will be discussed in a later section on context.

### Actors

An analysis of the actors involved in policymaking revealed that there is a strong collaboration between various relevant stakeholders. In all interviews, participants shared insight about their partnerships and consultations with a large diversity of stakeholders both internal and external to the government of Ontario.

#### External partners

External collaboration and consultations drive a lot of the work and decisions in the government of Ontario. For example, it is not infrequent for public servants to obtain expertise from research bodies, such as universities and research centres, outside of government to support their decision-making process. When working on files related to older adults and people living with disabilities, key concerns such as lack of data and privacy concerns with gathering sensitive data were the driver for external collaborations with agencies that have the data. In addition, external bodies such as research groups, not-for-profit organizations, advocacy organizations, health care professionals’ associations and their individual members, and people with lived experiences are often asked to participate in advisory roles to reflect on issues and provide recommendations. The main mechanism to engage with those groups is through the creation of permanent advisory bodies. These bodies are composed of a wide range of actors that give advice on issues of interest.

While collaborations with key groups via advisory bodies may be frequent, pan-Canadian collaborations with other provinces or territories seem to be less frequent and unstructured. They seem limited, at best, to knowledge exchange and sharing of best practices. P4 shared some examples of the informal information exchanges that would occur between them and other provinces:

Uhm and then provincial […]. I wouldn't say it impacts our work, but there is opportunity for collaboration and understanding lessons learned. Ontario being the biggest province, sometimes folks come to us, you know, for what are you doing, but also alternatively, some of the smaller provinces have some really great ideas and the capacity to implement those so it does very much go both ways, doesn't impact our day-to-day work, but there's definitely value in the partnership. (P4)

#### Internal partners

Internally, collaboration with diverse groups was portrayed as critical. Intradepartmental collaboration was frequent when expertise in a specific area was found in a different group of the government. For policy issues that concern older adults, internal collaboration among diverse sectors was noted throughout interviews. P7 indicated the necessity to keep internal channels open for communication and collaboration for policy issues concerning older adults specifically:

We as a division, as the Ministry, collaborate with all internal partner ministries, just given our stewardship. [We hold a] consultation role because most of the other ministries hold the levers of change, especially on the accessibility side. […] We have to make sure that we work with them to ensure that people with disabilities and seniors are considered in the policies, programs, initiatives that are being developed on a day-to-day basis. (P7)

Most frequently, participants mentioned that internal partners included individuals in policy areas where there was an obvious link (i.e., health and aging). They also confirmed the presence of multi-sectoral collaborations with other, more distant areas, but talked about those as being more ad hoc and less integrative.

### Contextual factors

Our study revealed that external pressures shape decisions and policy actions, and are derived from two main sources: the organization and structure of the Canadian healthcare system, and influences from unpredictable external pressures.

#### Organization and structure of the Canadian healthcare system

The Canadian system is organized using a federated system composed of 13 provincial and territorial (PT) governments and a Federal Government. In terms of health-related issues, the work (i.e., policies, program implementation and service delivery) falls in the jurisdiction of PT governments. However, the Federal Government has some strings attached with PT governments, usually because of funding-related mechanisms. In addition, the Federal Government usually convenes PT governments together around FPT Tables. For older adults’ self-management of disease and disability using ICTs, the Federal Government’s role varies. For example, home and community care isn’t an entitlement under Medicare which means that any policies related to self-management that fall under home and community care would not be mandated or involve the Federal Government. However, some recent indirect investments from the Federal Government for home and community care to provinces have forced linkages between the two levels of government. The investments came with some expectations where the Provincial Government had to meet the commitments outlined in an agreement with the Federal Government.

Federal-led forums have been pointed out by participants as a good avenue to get direction and collaborate across Canadian jurisdictions. For example, federal investments into home and community care have led to the creation of a pan-Canadian forum where provinces receive federal direction to make ensure alignment of their provincial initiatives. In addition, this forum has also allowed for the government of Ontario to share best practices with and gather lessons learned from other jurisdictions across Canada.

When specifically talking about technology, participants shared insights on the roles of the Federal Government in comparison to that of the PT. Participants viewed the Federal Government as having a role to play in strategizing on technology needs to support the population and regulating the facilities (private sector enterprise) where the technologies are created, while the provincial role is to ensure company business development from a market and growth perspective.

Furthermore, participants discussed the importance of provincial political agendas and their influence on the non-partisan work that they do. When a provincial government is elected, the goals and mandates set forward by the elected government officials have huge impact on the outcomes and activities that happen within the public service. Participants shared that the political pressure serves as the guidance for the work that they do as public servants.

Participants also pointed to the impact of government ideologies on investments in specific sectors. For example, the economic development priorities of the government in place during the conduct of interviews were heavily focussed on life sciences and job creation, especially in the medical technology field called MedTech. This focus was viewed by participants as an indirect support to health of the population and to older adults’ self-management of disease and disability using ICTs.

#### Influences from unpredictable external pressures

In all interviews, participants emphasized the impacts of unpredictable external pressures on policymaking. The main discussion point during all interviews was the influence of the COVID-19 pandemic on policy actions influencing older adults. For home care specifically, there was a push toward increasing and expanding virtual care supports due to the increased risk for in person consultations during the COVID-19 pandemic. P2 shared their experience in adapting current models of care because of the COVID-19 pandemic:

So we really, during COVID, expanded the use of virtual care in home and community care including friendly visits and that kind of thing. But beyond sort of permitting and facilitating the use of digital virtual visits, we have not done, from my division, we have not done more than that. […] During COVID, one of the expansions of the monitoring initiative was related to COVID at home program […]. It was the primary care initiative where they used the oxygen stat monitors and then had monitoring or oversights linkage with primary care for people who were sort of managing through or recovering from COVID at home. (P2)

The COVID-19 pandemic also led to many reflections about programming, especially for diverse groups of Ontarians. Participants mentioned specific challenges in ensuring continuity in programming for individuals living in northern areas for example, which often resulted in increased isolation. While challenges and difficulties were enumerated by participants, they also talked about the potential to act on them and find proper solutions moving forward.

One positive outcome that stemmed from the COVID-19 pandemic is an increase in the efficiency of processes. Participants mentioned that many government platforms were becoming electronic as a result of the digital push during the pandemic. For example, the Ontario social assistance program implemented an electronic document management system as a result of pressures from the COVID-19 pandemic. This finding specifically suggests the intersection between contextual factors and other factors of policymaking, such as processes.

### Summary

In summary, interviews with public servants from various ministries revealed that the development and deployment of ICTs targeting the self-management of disease and disabilities in older adults exists in various areas of policymaking. Legislation, regulations, programs and services, are affected by unpredictable circumstances, while being developed, implemented and evaluated through defined government processes and collaborations among various key actors.

## Discussion

The analysis of policies on older adults’ self-management of disease and disability using ICTs in Ontario revealed influences that shape the government’s approach to policymaking. The interviews with policymakers suggest that efforts are targeted toward (1) diverse groups including older adults, (2) people living with disabilities and (3) businesses. In addition, policymakers engage with policies through various mechanisms including funding programs or service delivery, and through legislation and regulation oversight.

Findings revealed that processes for developing these policies are based on four steps: (1) idea generation, (2) policy development (often intertwined with idea generation), (3) implementation, and (4) evaluation. These steps were expected as they align with the well-known policy cycle process [23]. As expected, the importance given for each of the steps, varied depending on the nature of the policy, where in the legislative and regulatory sphere, a larger emphasis is given to idea generation and policy development, while in the programming and service delivery sphere, a larger emphasis is placed on implementation and evaluation. Government of Ontario policies seem to be developed by a diversified group of actors that collaborate in the process. There are strong partnerships between internal and external stakeholders that are involved in different steps, but usually increasingly in the idea generation and policy development steps of the process. This level of collaboration was expected and confirmed by the interviews.

Finally, context has been demonstrated as having a large impact on policy decisions. The federated organization of the Canadian healthcare system (e.g. funding with “strings-attached” and knowledge exchange through FPT tables) and unpredictable external events (e.g. COVID-9 pandemic) all shape policy decisions on older adults’ self-management of disease and disability using ICTs. While we expected some external pressures to influence policy decision-making, the colossal impact of a global pandemic on forcing digitalization into policies was surprising.

As summarized above, the policy triangle [9] helped to discover the complexity of interactions between different components of policies on older adults’ self-management of disease and disability using ICTs. One major strength of the government of Ontario’s approach is the integration of dialogue among multiple levels and stakeholders to inform policymaking. As a health-dominated topic, government of Ontario policies on self-management of disease and disability using ICTs also span across distant fields such as accessibility, economy, business, and transportation. For example, participants from unintended domains such as the Ministry of Economic Development, Job Creation and Trade requested to participate as they felt their areas of work were related to the issue. However, participants from these quite different areas of work did not seem to work very closely with one another. The limited crossing between different policy areas portray policy efforts that are mostly additive rather than integrative, rendering the implementation to be lengthier. The different ministries advance individual files and projects that have an impact on older adults with chronic diseases and disability using ICTs without much cross-fertilization and integration. This could have some consequences for duplicating efforts toward a common goal, and therefore reduce efficiency in policymaking and efforts to achieve strategic orientations. Cross-sectoral policymaking poses some increased challenges, such as increased difficulties in coordination or increased risk of conflict between sectors, but the benefits it presents outweigh the difficulties [24]. When participants presented examples of cross-sectoral collaboration, the disciplinary overlap was more obvious, such as collaboration in sharing information and data between the Ministry of Health and the Ministry for Seniors and Accessibility. For example, home care and older adults are topics that should not be addressed independently from each other and work on those issues was said to be more collaborative and integrative by interviewees. Policymaking that use collaborative approaches align with previous research that stipulates that addressing chronic diseases require this outlook that allows to see the bigger picture of the issue at stake [25]. Moving forward, policymaking on the topic of self-management should continue to use collaborative approaches that span across sectors including those with more distant boundary overlaps. There could be increased efforts to move toward more collaborative dialogue in policymaking [26] where the multiple sectors of the provincial government interact with one another by establishing new networks and co-create solutions that ponder on the various areas involved. Co-creation is one tool that could be explored and offers promising benefits for policymaking [27]. In the co-creation of policies, explicit intersectoral involvement, between ministries for example, may lead to innovation and unique ways of designing and solving complex problems.

The policy triangle [9] also helped to identify challenges with policymaking within the government of Ontario. Policy efforts that support older adults’ self-management of disease and disability using ICTs were significantly impacted as result of the COVID-19 pandemic, an unpredictable external pressure. While the government of Ontario previously demonstrated few efforts to support self-management activities via ICTs [17], the COVID-19 pandemic has triggered a fundamental shift in digital health in various programs and services. With limited pre-pandemic efforts from the government of Ontario to include digital tools to support self-management, several barriers that are age-related and socio-cultural may have either positively or negatively impacted older adults who were left needing to adapt or failed to adapt to pandemic-generated digitalization [28]. The COVID-19 pandemic has demonstrated that the current system is mainly reactive rather than proactive regarding the implementation of digital solutions to support older adults’ self-management. Reactivity, however, may not be the adequate approach to implementing digital solutions for older adults as it risks leaving some behind due to pre-existing inequities. Recent literature suggests that public health policies should consider implementing solutions that are cognizant of eHealth literacy, intergenerational in nature, supported with adequate training and education, and culturally appropriate to sub-groups of older adults [29]. With a reactive approach, policymakers do not have time or sufficient people resources to ensure that policies effectively consider and address the various components highlighted above. Policymaking that is anticipatory in nature could be better suited for modernizing programs, services, legislation and regulations that relates to older adults’ self-management of disease and disability using ICTs. In fact, Tõnurist and Hanson [30] point to proactive policymaking as a good avenue for addressing complex and unpredictable policy questions. With rapid evolutions happening in the technological world, even in that of technologies that support older adults’ health, now called AgeTech [31], the government of Ontario would benefit from increased proactivity and action on this issue. From a broader perspective, actions at multiple levels including the Federal Government of Canada in collaboration with PT governments is needed to accelerate the inclusion of digital solutions into policy actions.

## Limitations

The study presents several limitations that should be considered in the interpretation of the research findings.

First, data collection focussed on a select group of government ministries because of their likelihood to address issues related to older adults, persons with disabilities, self-management, and technology. This could have led to the noninclusion of ministries that may have worked on polices related to the topic of interest. This was mitigated, to a certain degree, by allowing participants to refer the researchers to individuals in other ministries, through snowball sampling.

Second, this study used a single model for policy analysis. The policy triangle by Walt and Gilson [9] guided data collection as it structured the interview format and formed the basis for data analysis via the deductive coding structure. This model was the most appropriate to ensure that several components of policymaking be considered in the analysis of policies. As stated in Walt and Gilson [9], other models for policy analysis exist, but largely focus on evaluating content of policies alone. Walt and Gilson’s [9], policy analysis model revealed the strength of holistic analysis of policies and the requirement to consider factors such as the content of policies, the actors involved in policy development, implementation and evaluation, the process of policymaking and the context surrounding policymaking.

Third, data collection occurred during the COVID-19 pandemic and just before a provincial political election. Both these external factors could have resulted in a decrease participation from policymakers working through various competing priorities. While 59 public servants were invited, only a total of 10 accepted to participate. During the exploration of contextual factors impacting policymaking, participants put the COVID-19 pandemic at the center of discussions. While COVID-19 had huge effects on policies within the last 2 years, this could have detracted participants from discussing other contextual pressures that were present a priori.

## Future research

Future research should engage policymakers in the development of solutions to mitigate challenges and limitations of policies on older adults’ self-management of disease and disability using ICTs. In doing so, the solutions discussed would better reflect needs and capacity within the current policy environment. These would include looking for ways to make the system less reactive and more proactive in keeping up with innovations and advancements in ICTs to support older adults’ self-management. The COVID-19 pandemic, which has forced digitalization considerations and integration of digital solutions into policies, revealed a readiness of the system to move into these directions.

In addition, future research could investigate the outcomes and results of reactive policies that have been put in place during the COVID-19 pandemic. This would help to better understand the impacts on diverse groups of older adults in Ontario. It may also point to whether changes need to be made and if other avenues such as foresight and futures-thinking may be relevant tools to anticipate and make predictions about future concerns.

### Take-home messages

- Polices regarding older adults’ self-management of chronic diseases and disabilities using ICTs exist as programs, services, legislation, and regulations, where some technology components are embedded. However, the government of Ontario lags in the digital modernization of policies in comparison to the pace of technology advancement and development.
- A variety of ministries of the government of Ontario, even those with unusual ties to health, are concerned with and working toward improving older adults’ self-management of disease and disability using ICTs. Some ministries are working closely with one another and others acting more independently to address issues in this sphere.
- Context, in the form of external unpredictable pressures, have a tremendous impact on moving government actions toward digitalization of programs and services for older adults. This demonstrates a reactive rather than proactive policymaking system.

## Supplementary Information

**Additional file 1.** Semi-structured Interview Guide. Semi-structured interview guide which lists all questions that guided the interviews with study participants.

## Data Availability

The datasets used and/or analysed during the current study are available from the corresponding author on reasonable request.

## Abbreviations

ICTs: Information and Communication Technologies
FPT: Federal, Provincial and Territorial
AODA: Accessibility for Ontarians with Disabilities
OTN: Ontario Telemedicine Network
PT: Provincial and territorial
NCE: Networks of Centres of Excellence
