# Supplementary material for: Policymaker perspectives on self-management of disease and disabilities using information and communication technologies
Source: Health Res Policy Syst. 2023 Jun 14;21:52. doi: 10.1186/s12961-023-01004-7 (PMC10264873; doi:10.1186/s12961-023-01004-7)
Supplement: Supplementary file 1 — Additional file 1. Semi-structured Interview Guide. Semi-structured interview guide which lists all questions that guided the interviews with study participants. [file 12961_2023_1004_MOESM1_ESM.pdf]

Semi-structured Interview guide

Section A – Background

1. What are the main files that you work on? (if no answer, hesitation or a no because it is confidential) What were files you previously worked-on and that have been implemented (or not, discarded and why) ?
2. What are the main files that your team/division work on?
3. More specifically, in terms of older adults self-management of disabilities using information and communication technologies:
    a. Can you expand on the nature of the work that you do in that area
    b. What policy documents (if any) have you developed in the area of self-management specifically?
        i. Are these documents publicly available? Where can they be accessed?
4. Could you please describe what you do on a day-to-day basis?
5. Which teams do you collaborate with? Intra vs inter-deparments
    a. [If they collaborate with other teams] What is the nature of this collaboration? Legal, regulatory, budgetary
        i. How often are you meeting with other teams?
        ii. Who is more involved in decision-making?
        iii. Who leads this collaboration?

Section B – Political Context

1. To what extent do other provincial or federal initiatives guide or drive the work that you do? What about municipal work?

2. How is the provincial political agenda impacting the work that you do? (Influence of the election cycle, gov. political parties' view, etc.)

Section C – Policies (Note: take good notes of the titles and try to identify web links)

1. Does your division develop or oversee any policies?
    a. [If yes] What policies did your division develop or oversees?
2. Who decides which policies are developed/overseen by your division?
3. Who is impacted by the policies that you develop/oversee?
4. What is the process for designing and implementing policies in your division?

Section D – Programs (Note: Pay attention to details – ask if there are joint programs at the municipals or federal levels and co-funding)

1. Does your division deliver and oversee any programs?
    a. [If yes] What programs does your division deliver or oversee?
    b. [If yes] Who decides how these programs are delivered/overseen?
    c. [If yes] Who is involved in these programs? (Other divisions, community organizations, others?)
    d. [If yes] Who are the beneficiaries of programs you deliver?
2. What is the process for designing, delivering and overseeing programs in your division?
    a. Design:
        i. Who decides that a program need to be designed?
        ii. Who designs the programs?
        iii. Who gives funding for the programs?

    iv. What is the core origin of the program ? New government, from within the division ?

  b. Delivery:

    i. Who delivers the program?

    ii. Who mostly benefits from the programs?

    iii. Who makes decisions on how the program is delivered? Be prepared to give examples to any questions – ex. political

  c. Oversight:

    i. Who assesses the impact of a program? What is the process – review who is engaged, every year, 2 years, election cycle

    ii. Who ensures that the program is delivered according to its initial intent? Deliverables vs within budget

Section E – Service Delivery

1. Does your division deliver any services to the community?
    a. [if yes] What services does your division deliver?
    b. [If yes] To whom does your division deliver services?
    c. [If yes] For what purpose does your division deliver services?
2. What is the decision process for delivering services? What qualifies people or organizations to get the funding and then deliver them
3. How are service deliveries managed by your organization? Yearly, budget cycle, more tight control

Section F - Innovation

1. What is your definition of innovation ?
2. What is the importance and role of innovations in the development of policies and /or programs that have been developed or implemented by your division?
3. Describe how (the process) innovations get adopted in your division? Maybe ask for the initial source
4. How does your division keep “up to date" with recent innovations in the realm of aging and technology or disability and technology?
5. What is the role of public servants in innovating on a day-to-day basis?
6. What is the approval process for suggesting innovations in processes/policies/programs etc.?
